# Autopsy of the Hon. Daniel Webster

**Published:** 1853-03

**Authors:** 


*Autopsy of the Hon. Daniel Webster.*—[From an article in the American Journal of Medical Sciences, we extract the following description of the post-mortem appearances, &c., by his physician, Dr. John Jeffries.]

The autopsy was made by Dr. J. B. S. Jackson, who furnishes the following report:

*Autopsy* thirty-two hours after death; present Drs. Jeffries, Porter, J. Mason Warren, Wyman, Parkman, and Jackson.

The emaciation was very marked, as shown by the state of the integuments and muscles; the latter being wasted, pale, and flabby.

*Abdomen.*—The peritoneal cavity contained eleven pints of serum. There were also old and strong adhesions about the spleen, the gall-bladder, the cæcum, and to a small extent between the left extremities of the arch of the colon and the parietes of the abdomen.

The stomach was distended, and contained half a pint of very dark blood, about one half of which was in the state of a soft coagulum; and this was the only appearance that was found of coagulum in any part of the body. The mucous membrane was deeply stained by the contents, generally rather soft, and in the pyloric portion somewhat mamellonated. The intestines were opened throughout, washed, and fully examined with reference to the diarrhœa that had so long existed. Blood was found throughout in very considerable quantity as far as the descending colon, below which there was no trace of it; in the large intestine it was altered as usual in color. Mucous membrane stained by the contents so far as blood extended. In the large intestine were numerous herniæ of the mucous membrane, so common in this situation; from many of these, small masses of feces or of mucus could be forced out, and these were the only traces of feces that could be found. Otherwise the mucous membrane of the intestines appeared quite healthy; there being nowhere any ulceration to explain the diarrhœa, nor ecchymosis connected with the hemorrhage.

The liver was throughout very markedly granulated; dense, and contracted in size; the color externally was greenish or bronzed, but internally everywhere of a pale red; showing, as we may not very unfrequently observe, the inappropriateness of the term "cirrhosis," which would generally have been applied to the present case. Weight of the organ, three pounds and one-third, avoirdupois. Bile in the gall-bladder nearly black, and of a tarry consistence.

Spleen small, pale, and shriveled. Investing membrane to some extent opaque, white, thickened, and condensed; this change being probably due to the old peritoneal affection.

Kidneys and pelvic organs healthy.

*Thorax.*—Old pleural adhesions over nearly the whole of the right side; none on the left. Lower lobe of the left lung and the two lower lobes of the right much congested, and very dark; a change that undoubtedly occurred toward the close of life, being simply passive.

Heart flaccid; very little blood in cavities, and this was quite liquid. Slight disease of aortal valves, but organ otherwise healthy. Foramen ovale; a small valvular opening existed. Aorta not ossified except to a small extent in the abdomen.

*Head.*—The membranes of the brain were most remarkably diseased. In the cavity of the arachnoid was a layer of fibrine which covered almost entirely, and about equally, the convexity of both hemispheres; It did not extend, however, beneath nor between them, nor about the cerebellum. In the recent state, it had a rather dull, yellowish, infiltrated, œdematous appearance; being one-fourth of an inch in thickness over the upper surface, but becoming gradually more thin on the sides, where it terminated in a thin edge. The adhesion to the dura mater was in some parts quite close; but it was generally very readily stripped off, and left the arachnoid with its usual polish. It was more adherent to the subjacent membrane; this last being irregular, and having generally a clouded and slightly opaque appearance, with many milk-white spots, but without any appreciable thickening. The quantity of serous effusion into the membranes was altogether large. The subarachnoid tissue, corresponding to the layer of fibrin above described, was infiltrated with a straw-colored serum in some places, separating the convolutions from each other; this separation was quite remarkable at the posterior part of the right cerebral hemisphere on its upper surface and near the median line, there being also a slight depression at this part. The dura mater adhered firmly to the calvaria, but was healthy in structure, as were the membranes otherwise; there was, however, a serous infiltration into each plexus choroides; though no more, if not less than usual, into the lateral ventricles. No appearance of recent meningitis; and no effused blood or cysts in or about the false membrane. The brain itself was perfectly healthy; and the arteries at the base very nearly so. Cranium healthy. Over the right frontal region a scar existed, the result of the injury that occurred last May; integuments not otherwise remarkable.

A portion of the fibrine from the arachnoid cavity having been removed for microscopical examination, it was found, some hours afterward, and when the serum with which it had been infiltrated was absorbed, to have almost the consistence of one of the natural tissues of the body; being strong enough to bear considerable traction; it also appeared then to have somewhat of a laminated structure, and blood-vessels were distinctly seen in it, even with the naked eye. Dr. Wyman found it "organized, and, in some places, vascular. Under the microscope, the lymph was resolved into minute fibers, like those forming the white fibrous element of areolar tissue, and including in their meshes large numbers of minute granules."—*N. York Med. Gaz.*

